# Case Report: The diagnostic and therapeutic crossroads: when myelofibrosis transforms into mixed phenotype acute leukemia

**DOI:** 10.3389/fonc.2026.1811409

**Published:** 2026-03-31

**Authors:** Yanquan Liu, Jingdong Zhang, Xiangzhou Du, Lihong Liao

**Affiliations:** Department of Hematology, Ganzhou People’s Hospital (The Affiliated Ganzhou Hospital of Nanchang University), Jiangxi Health Commission Key Laboratory of Leukemia, Ganzhou, Jiangxi, China

**Keywords:** diagnostic challenge, elderly patient, leukemic transformation, mixed phenotype acute leukemia, myelofibrosis, prognosis, therapeutic dilemma

## Abstract

**Background:**

Myelofibrosis (MF) is a myeloproliferative neoplasm characterized by the proliferation of fibrous tissue in the bone marrow. Transformation to acute leukemia, typically acute myeloid leukemia, represents a terminal event in its natural history; however, transformation to mixed phenotype acute leukemia (MPAL) is exceedingly rare. The diagnosis and treatment of MPAL are particularly challenging, especially in elderly patients with underlying MF. The clinical characteristics, treatment response, and prognosis of such cases remain unclear and are rarely reported.

**Case presentation:**

This article presents a retrospective analysis of a 75-year-old female patient who initially presented in October 2023 with splenomegaly and thrombocytopenia. Based on bone marrow morphology, biopsy (revealing Grade MF-2 fibrosis), and molecular testing (detecting a *JAK2* ^V617F^ mutation with a variant allele frequency of 44.67% and an *ASXL1* mutation), a diagnosis of primary myelofibrosis (PMF) was established. The patient did not receive standard targeted therapy and was subsequently lost to follow-up. Approximately two years later (September 2025), she was readmitted due to a significantly elevated white blood cell count (47.14 × 10^9^/L) with 64% blasts in the peripheral blood. A comprehensive re-evaluation using MICM (Morphology, Immunology, Cytogenetics, Molecular) classification was performed: bone marrow flow cytometry revealed that 65.73% of the blasts co-expressed CD34, CD117, and the T-lineage marker CD7. Molecular testing detected the persistence of the original *JAK2* and *ASXL1* mutations, with the acquisition of additional *NRAS*, *EZH2*, and *TET2* mutations. A definitive diagnosis of MPAL (T/Myeloid mixed phenotype) transformed from PMF was established. Given the patient’s advanced age and underlying MF, two cycles of a low-intensity chemotherapy regimen primarily based on the “VP regimen (Vincristine + Prednisone) combined with Azacitidine” were administered. Initial treatment resulted in a complete remission with incomplete hematologic recovery (CRi). Considering the incomplete recovery of peripheral blood platelets and the presence of minimal residual blasts detected by bone marrow flow cytometry, the patient was started on a regimen combining the VP regimen with a VA regimen chemotherapy to achieve a deep and durable complete remission. As of the latest follow-up, the patient’s relevant laboratory results remain within a safe range, with no peripheral blasts detected. Her psychological and physiological status is acceptable, she reports no specific discomfort, and her disease is well-controlled.

**Discussion:**

This rare case offers several critical insights: 1) Rare Transformation Type: It clearly delineates the complete clinical and molecular evolutionary trajectory of PMF transforming into the rare MPAL, underscoring the importance of precise immunophenotyping during the blast phase of MF to identify atypical transformations. 2) Clonal Evolution Pattern: The molecular profile evolved from typical MPN driver mutations (*JAK2*, *ASXL1*) to the acquisition of additional mutations associated with leukemic transformation (*NRAS*, *EZH2*, *TET2*), providing a concrete example for understanding the genetic mechanisms underlying such transformations. 3) Treatment Dilemma in the Elderly: The case highlights the immense therapeutic challenges in elderly MPAL patients with concomitant persistent myelofibrosis. While low-intensity regimens may induce short-term remission, the responses are not durable. Patient tolerability to intensive chemotherapy and novel targeted agents (e.g., Venetoclax) is poor, leading to a dismal prognosis. 4) Management Implications: It emphasizes the necessity of long-term, regular follow-up and early intervention for MF patients, particularly those harboring high-risk mutations. Furthermore, it underscores the urgent need for more effective and better-tolerated novel therapies for this patient population.

**Conclusion:**

Transformation of MF to MPAL is a rare event associated with an poor prognosis. This study provides the detailed report of an elderly patient with MPAL arising from *JAK2*-positive PMF. This case not only serves as a unique model illustrating the complex evolution of a malignant clone but also profoundly reveals the unique therapeutic challenges and extremely poor survival outcome resulting from the convergence of advanced age, MF background, and MPAL transformation. It offers pivotal real-world evidence for the clinical management of this specific patient population and highlights the need to explore novel therapeutic strategies.

## 1 Introduction

Myelofibrosis (MF) is a Philadelphia-negative myeloproliferative neoplasm (MPN) characterized by clonal myeloproliferation, reactive bone marrow fibrosis, splenomegaly, and constitutional symptoms (1, 2). Its natural history is often marked by disease progression, with transformation to acute leukemia representing a terminal event occurring in approximately 10-20% of patients (3, 4). While such transformations typically manifest as acute myeloid leukemia (AML), the evolution into mixed phenotype acute leukemia (MPAL) is exceptionally rare (5, 6).

MPAL itself is a heterogeneous group of acute leukemias where blasts display differentiation along more than one lineage—lymphoid and myeloid—co-expressing markers of different lineages without meeting the criteria for a specific entity (7). The diagnosis and management of MPAL are inherently challenging due to its biological complexity, lack of standardized therapeutic protocols, and generally poor prognosis (8, 9). These challenges are magnified in elderly patients with significant comorbidities, where tolerance to intensive chemotherapy is limited. The coexistence of an underlying myelofibrosis background further complicates the clinical picture, as it not only obscures diagnostic interpretation but also profoundly impacts bone marrow reserve and the safety and efficacy of subsequent therapies. To date, clinical features, optimal treatment approaches, and outcomes for elderly patients presenting with MPAL transformed from MF remain poorly defined.

Herein, we report a rare, unique and instructive case of a 75-year-old female with *JAK2*-positive primary myelofibrosis (PMF) who, after a period of suboptimal follow-up, presented with rapid progression to MPAL of T/Myeloid mixed phenotype. The transformation was accompanied by the acquisition of additional mutations (*NRAS*, *EZH2*, *TET2*), illustrating a complex clonal evolution. We detail the diagnostic journey, the therapeutic challenges encountered with low-intensity chemotherapy and targeted agents in the context of persistent myelofibrosis and advanced age, and the patient’s clinical course leading to a sustained, though incomplete, remission. This case provides critical insights into the rare phenomenon of MF-to-MPAL transformation, highlights the pitfalls in managing such high-risk elderly patients, and underscores the pressing need for more effective and tolerable therapeutic strategies for this distinct population.

## 2 Case presentation

**Figure 1:**
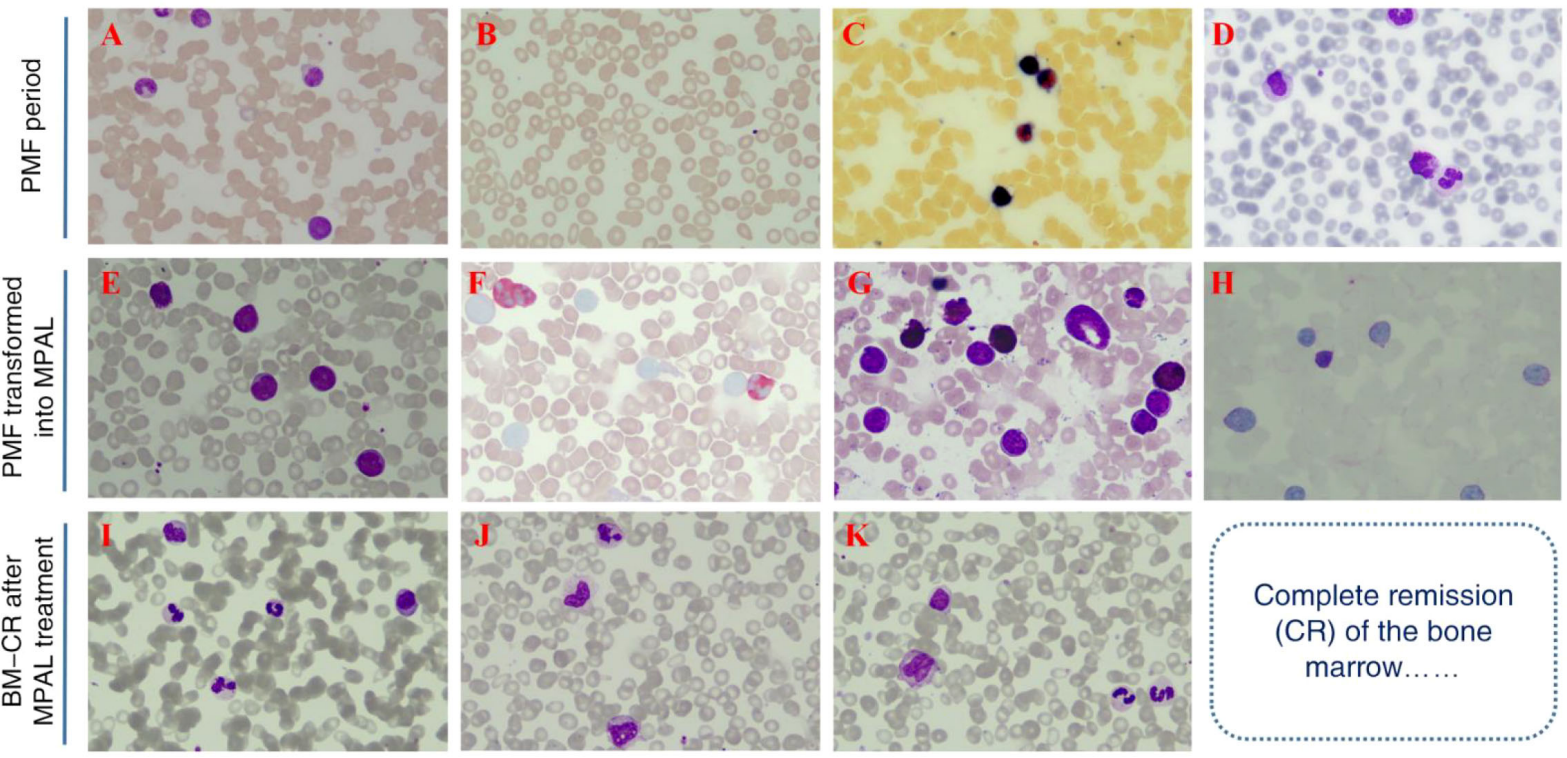
Bone marrow morphological staining of the patient. [**(A-D)** Results from the first bone marrow aspiration performed in October 2023. **(E-H)** Results from the second bone marrow aspiration performed in September 2025, suggestive of mixed phenotype acute leukemia. **(I-K)** Bone marrow results obtained in December 2025 after three cycles of chemotherapy, showing achievement of complete remission [CR]).

**Figure 2:**
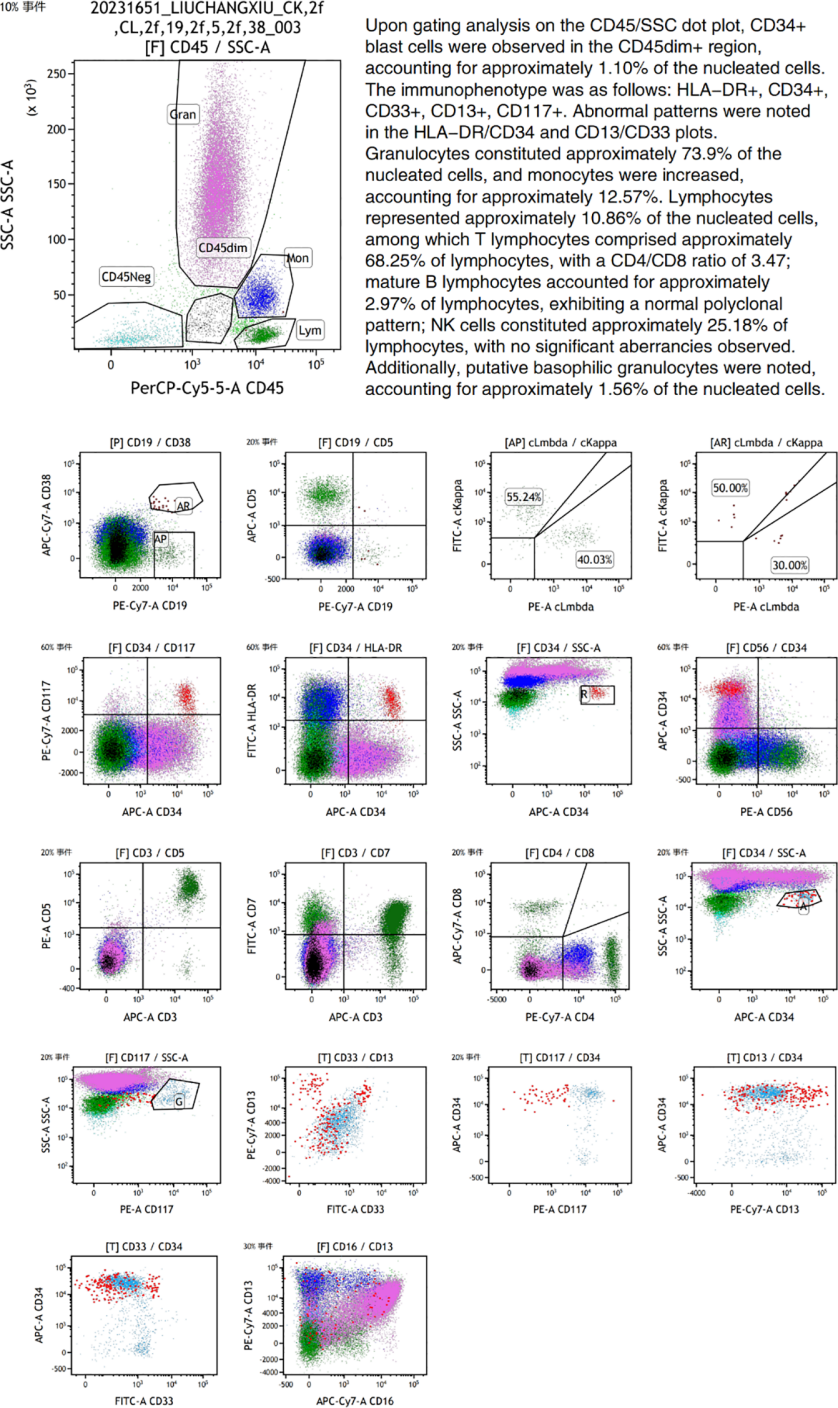
Bone marrow flow cytometric immunophenotyping results from October 2023. (Notes: Acquisition and analysis of 300,000 events were performed using a BD FACSCanto II flow cytometer. The gating strategy employed was CD45/SSC. A six-color antibody panel was utilized. Acquisition parameters included FSC-Lin, SSC-Lin, FL1-Log, FL2-Log, FL3-Log, FL4-Log, FL5-Log, and FL6-Log. The following antigens were assessed: CD3, CD7, CD45, CD5, CD56, HLA-DR, CD34, CD117, CD19, CD4, CD8, CD38, Kappa, Lambda, CD16, CD33, CD13).

**Figure 3:**
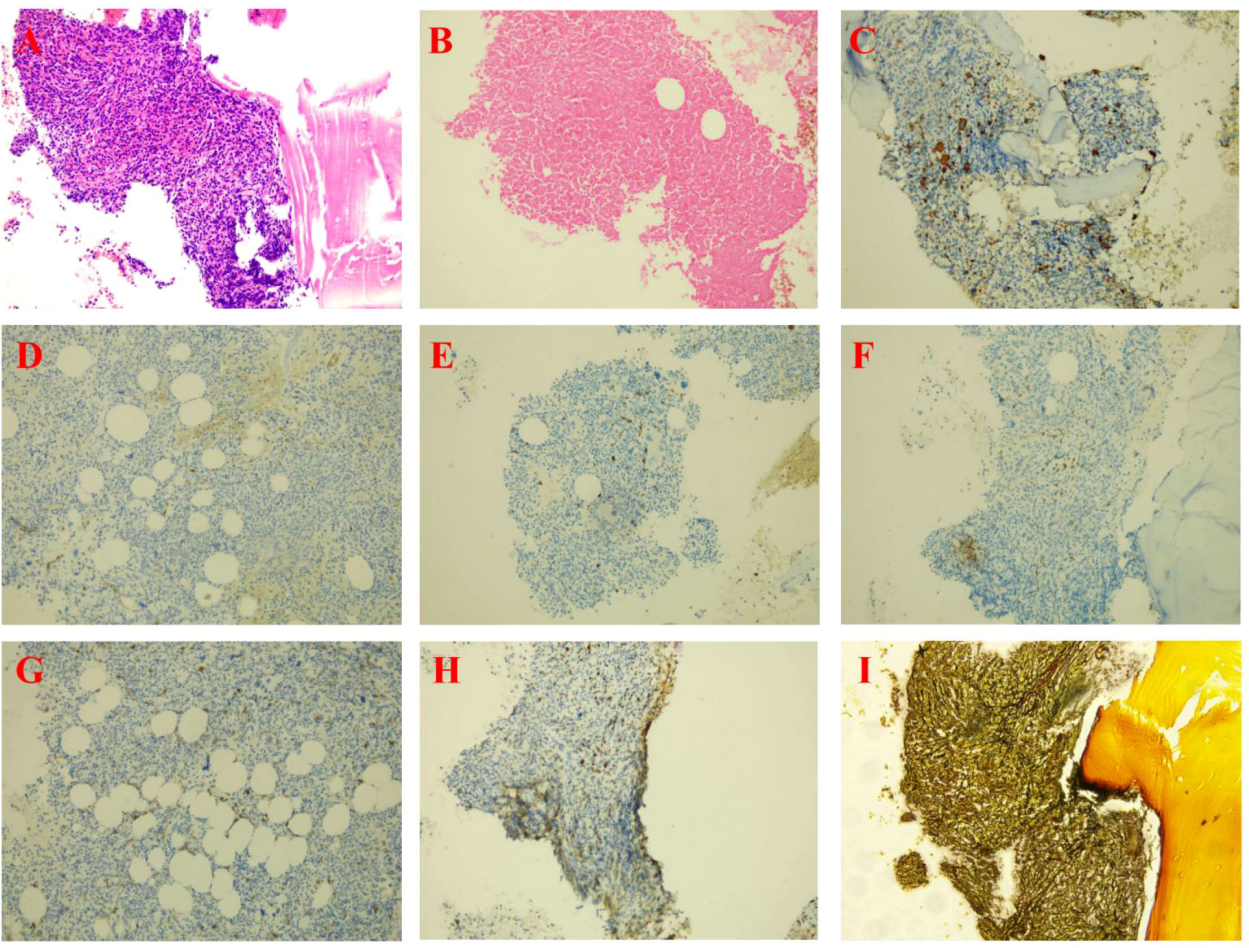
Bone marrow histopathological findings from October 2023. [**(A)** Hematoxylin and eosin [H&E] staining; **(B)** Iron [Fe] staining; **(C)** CD61 immunohistochemistry [IHC]; **(D)** CD34 IHC; **(E)** CD20 IHC; **(F)** CD3 IHC; **(G)** CD117 IHC; **(H)** Ki67 IHC; **(I)** Reticulin stain. All images are shown at 20× magnification].

The 75-year-old female patient, first presented to our hospital on October 7, 2023, with a chief complaint of “thrombocytopenia and splenomegaly for one month.” Her past medical history was significant for lumbar surgery. Physical examination on admission revealed splenomegaly, measured as: Line I: 9 cm; Line II: 11.5 cm; Line III: -1.5 cm. Initial Laboratory Findings (October 7, 2023): Complete Blood Count (CBC): White blood cell (WBC) count: 16.66 × 10^9^/L, Red blood cell (RBC) count: 3.85 × 10¹²/L, Hemoglobin (Hb): 110 g/L, Platelet (PLT) count: 40 × 10^9^/L, Reticulocyte count: 65.80 × 10^9^/L. Blood biochemistry: Total bilirubin: 15.36 µmol/L, Direct bilirubin: 4.83 µmol/L, Indirect bilirubin: 10.53 µmol/L, Alanine aminotransferase (ALT): 19.03 U/L, Aspartate aminotransferase (AST): 25.01 U/L, Total protein: 83.40 g/L, Albumin: 44.48 g/L, Globulin: 38.92 g/L, Urea: 4.04 mmol/L, Creatinine: 72.18 µmol/L, Uric acid: 430.14 µmol/L, Potassium: 3.72 mmol/L, Sodium: 136.74 mmol/L, Chloride: 106.33 mmol/L, Calcium: 2.33 mmol/L, Magnesium: 0.77 mmol/L, Lactate dehydrogenase (LDH): 378.64 U/L, Creatine kinase (CK): 30.74 U/L, CK-MB: 7.85 U/L, α-Hydroxybutyrate dehydrogenase (HBDH): 288.55 U/L, Vitamin B12: 643.86 pg/mL, Ferritin: 38.66 ng/mL, Folic acid: 7.12 ng/mL, Erythropoietin: 7.9 IU/L. Peripheral Blood Morphology: Blasts: 1%, Myelocytes: 2%, Metamyelocytes: 5%, Band neutrophils: 10%, Segmented neutrophils: 51%, Eosinophils: 1%, Basophils: 2%, Mature lymphocytes: 11%, Mature monocytes: 17%. Total WBC count was increased. Occasional blasts were observed. No nucleated RBCs were seen per 100 WBCs counted. RBCs showed anisocytosis. Platelets were sparse, individually distributed, with some large forms noted. Bone marrow aspiration and biopsy were performed on the day of admission (October 7, 2023). The bone marrow morphology report from October 8, 2023 (**Figure 1A**) suggested myelofibrosis, with a myeloproliferative neoplasm (MPN) not excluded; correlation with *JAK2* and other genetic tests was recommended. The bone marrow immunophenotyping report (**Figure 2**) indicated: CD34+ blasts accounting for approximately 1.10% of nucleated cells, with abnormal patterns observed in the HLA-DR/CD34 and CD13/CD33 plots. The bone marrow biopsy pathology report from October 11, 2023 (**Figure 3**) showed: H&E and PAS staining revealed extensive fibrous tissue proliferation in the bone marrow. The myeloid-to-erythroid ratio was increased. The myeloid lineage was present at all stages, predominantly myelocytes and more mature forms, with an increase in eosinophils. The erythroid lineage was present at all stages, predominantly late erythroblasts. Megakaryocytes were numerous, variable in size, with an increased nuclear-to-cytoplasmic ratio, hyperchromatic nuclei (spherical or irregularly folded), and formed dense clusters. Lymphocytes and plasma cells were scattered. Reticulin stain demonstrated Grade MF-2 fibrosis. The pathological conclusion for the bone marrow biopsy was: Mild left shift of the myeloid lineage with megakaryocyte morphological abnormalities and fibrous tissue proliferation in the bone marrow; correlation with clinical and genetic findings was recommended to differentiate primary from secondary myelofibrosis. Immunohistochemistry results showed: CD117 (partial +), CD235a (+), CD34 (-), CD20 (clone L26) (-), CD79a (-), CD61 (+), CD3 (-), MPO (+), Lysozyme (+), Ki67 (20%+). The cytogenetic (chromosome) examination showed no significant abnormal results. Furthermore, molecular analysis was performed, revealing: *JAK2 ^V617F^* mutation (Q-PCR) copy number: 10,674 copies; *JAK2* wild-type copy number: 13,218 copies; mutant allele burden: 44.676%. The genetic test indicated *JAK2 ^V617F^* (Q-PCR) copy number: 10,674 copies; *JAK2* wild-type copy number: 13,218 copies; mutant allele burden: 44.676%. Therefore, based on the comprehensive MICM (Morphology, Immunology, Cytogenetics, Molecular) classification, our team diagnosed the patient with Primary Myelofibrosis (*JAK2*-positive). Unfortunately, due to financial constraints, the patient declined targeted therapy with ruxolitinib and opted for treatment with oral lenalidomide 25 mg. After discharge, the patient also did not adhere to the recommended regular follow-up visits.

**Figure 4:**
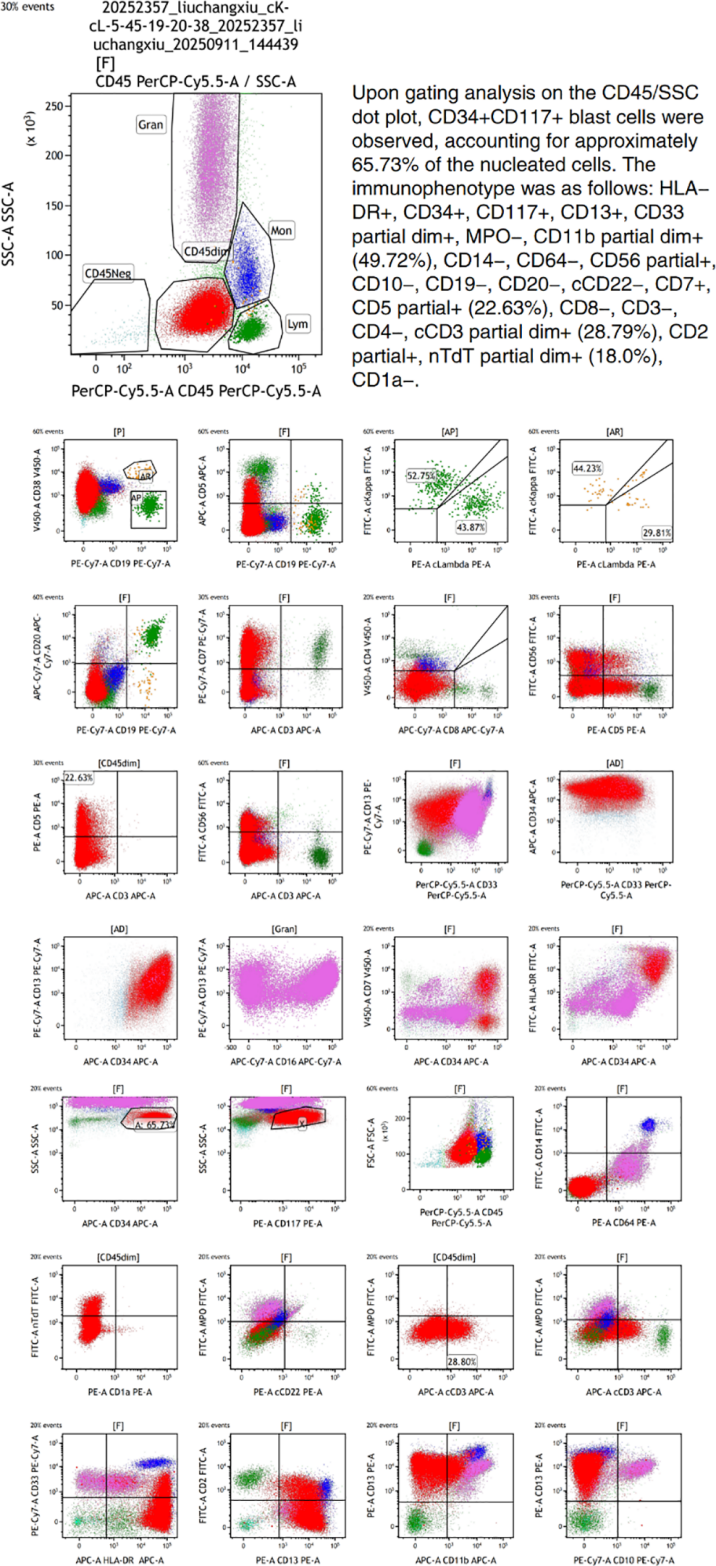
Bone marrow flow cytometric immunophenotyping results from September 2025. Acquisition and analysis of 300,000 events were performed using a BD FACSCanto II flow cytometer. The gating strategy employed was CD45/SSC. A six-color antibody panel was utilized. Acquisition parameters included FSC-Lin, SSC-Lin, FL1-Log, FL2-Log, FL3-Log, FL4-Log, FL5-Log, and FL6-Log. The following antigens were assessed: CD3, CD7, CD45, CD5, CD56, HLA-DR, CD34, CD117, CD19, CD4, CD8, CD38, Kappa, Lambda, CD16, CD13, CD33, CD7, CD20).

**Figure 5:**
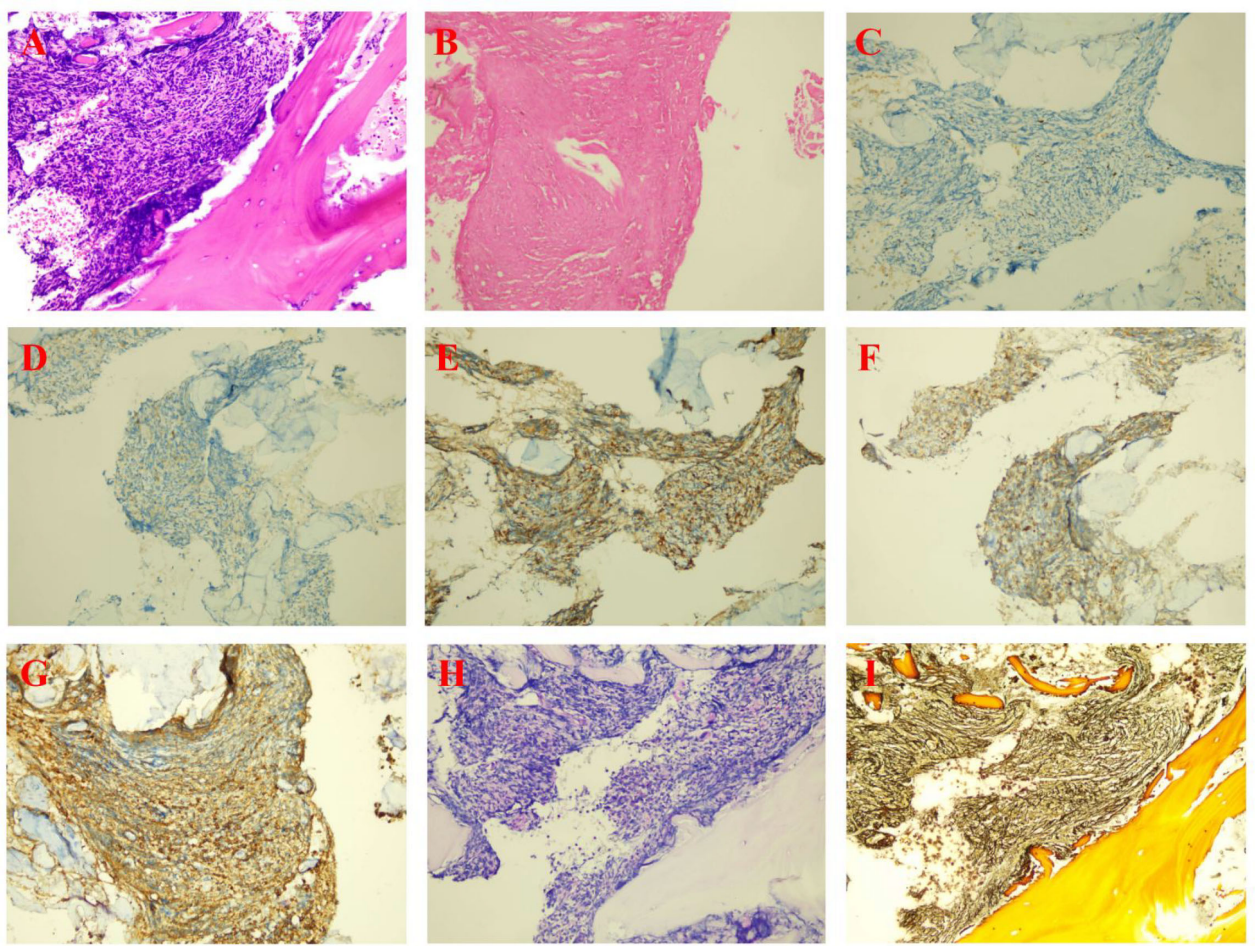
Bone marrow histopathological findings from September 2025. [**(A)** H&E staining; **(B)** Fe staining; **(C)** CD3 IHC; **(D)** CD4 IHC; **(E)** CD7 IHC; **(F)** CD34 IHC; **(G)** CD117 IHC; **(H)** Periodic acid-Schiff [PAS] staining; **(I)** Reticulin stain. All images are shown at 20× magnification].

**Figure 6:**
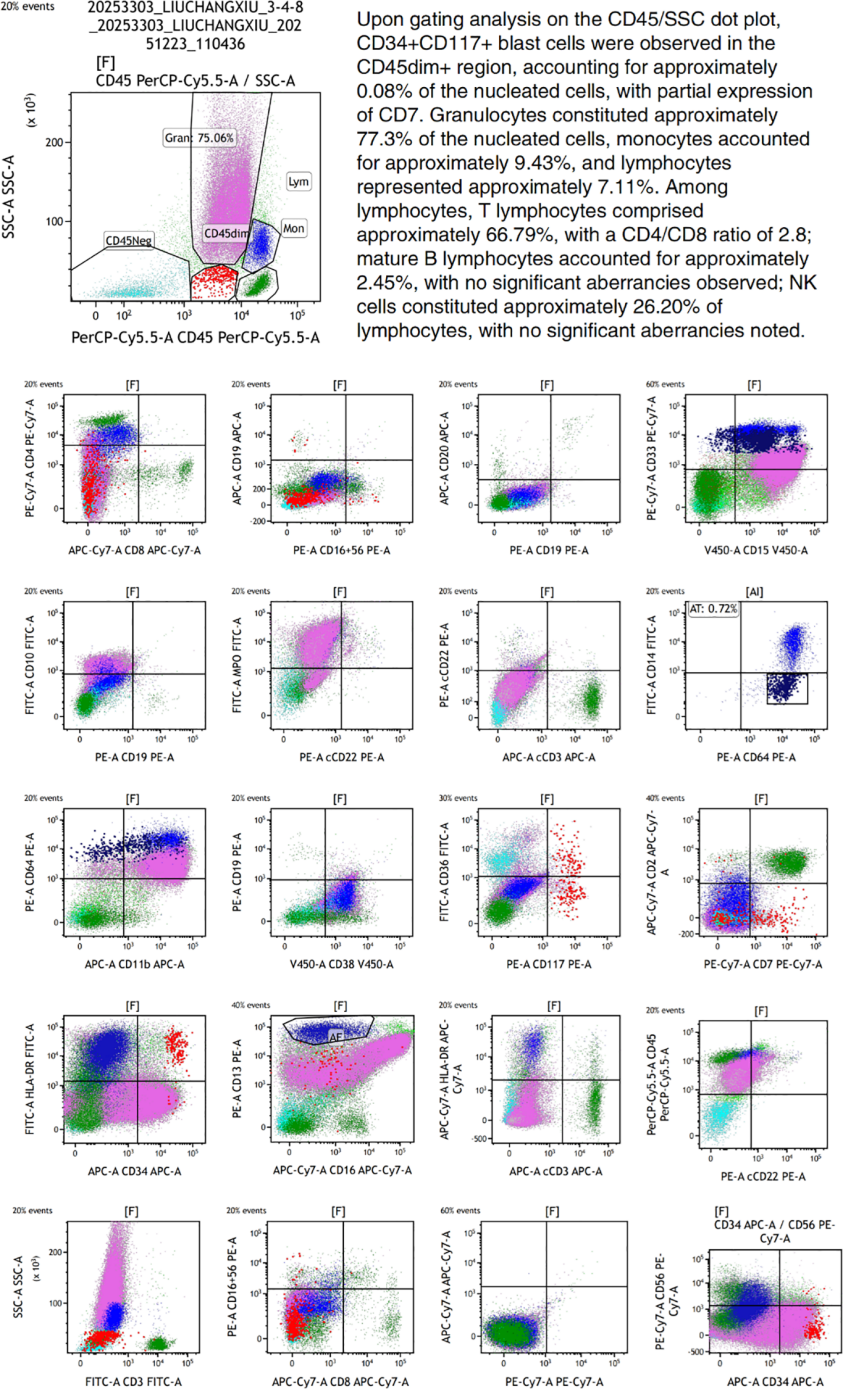
Bone marrow flow cytometric immunophenotyping results from December 2025. Acquisition and analysis of 100,000 events were performed using a BD FACSCanto II flow cytometer. The gating strategy employed was CD45/SSC. A four-color antibody panel was utilized. Acquisition parameters included FSC-Lin, SSC-Lin, FL1-Log, FL2-Log, FL3-Log, and FL4-Log. The following antigens were assessed: MPO, CD22, CD3, CD7, CD117, CD45, CD5, CD14, CD64, CD33, HLA-DR, CD56, CD34, CD2, CD13, CD11b, CD10, CD19, CD20, CD4, CD8).

On September 11, 2025, the patient was readmitted due to leukocytosis detected during a health examination. The following tests were performed: Complete Blood Count: WBC: 47.14 × 10^9^/L, Monocyte percentage: 59.5%, Absolute monocyte count: 28.06 × 10^9^/L, Hb: 131 g/L, PLT: 41 × 10^9^/L. Peripheral blood morphology for abnormal cells: Blasts: 64%. Blood chemistry indicated: Albumin: 35.71 g/L, Potassium: 2.91 mmol/L, Sodium: 136.40 mmol/L, LDH: 423.6 U/L. Given the abnormal blood counts and biochemical indicators upon this admission, our team performed another bone marrow aspiration and biopsy. Subsequently, the bone marrow morphology report from September 12, 2025 (**Figure 1B**) indicated: Suggestive of acute myeloid leukemia, non-M3 subtype. The bone marrow immunophenotyping report (**Figure 4**) revealed CD34+CD117+ blasts accounting for approximately 65.73% of nucleated cells, with predominant expression of T-lymphoid and myeloid markers. Furthermore, the bone marrow biopsy pathology report (**Figure 5**) showed: H&E and PAS staining revealed extensive fibrous tissue proliferation in the bone marrow; reticulin stain demonstrated Grade MF-2 fibrosis; myeloid left shift with megakaryocyte morphological abnormalities and fibrous tissue proliferation, consistent with acute myeloid leukemia with aberrant CD7 expression. Special staining results showed: Fe stain (-), Reticulin stain (+), PAS (+). Immunohistochemistry results showed CD117 (+), CD34 (+), CD3 (+), CD7 (+), CD8 (-), CD4 (-). Concurrently, molecular analysis for gene mutations and fusions was performed, revealing: Somatic SNV/Indel Class I variants: NRAS (p.G12S); Somatic SNV/Indel Class II variants: *ASXL1* (p.Q708*), *JAK2* (p.V617F); Somatic SNV/Indel Class III variants: *EZH2* (p.R658G), *TET2* (p.P980S). Therefore, based on the MICM classification, our team diagnosed the patient with Mixed Phenotype Acute Leukemia (MPAL). Initial cytoreductive therapy was administered with hydroxyurea 1g tid and dexamethasone 5mg for tumor lysis prophylaxis. Subsequently, on September 21, 2025, chemotherapy with the VP regimen combined with Azacitidine was initiated, specifically: Vincristine 1.4 mg/m² weekly + Prednisone 1 mg/kg + Azacitidine 100 mg on days 1-7. A bone marrow re-examination on October 31, 2025, showed: Bone marrow morphology indicated hypocellularity with 2.5% blasts; bone marrow immunophenotyping revealed CD117^+^CD34^dim+^ blasts accounting for approximately 8.25% of nucleated cells. The second cycle of chemotherapy was subsequently delayed. Another bone marrow re-examination on November 16, 2025, showed: The bone marrow morphology report indicated markedly active cellularity with 0.50% blasts. The bone marrow immunophenotyping report revealed residual abnormal CD34+CD117+ blasts accounting for approximately 0.30% of nucleated cells. CBC showed: WBC: 10.28 × 10^9^/L, Hb: 68 g/L, PLT: 45 × 10^9^/L. Disease status was assessed as complete remission with incomplete hematological recovery (CRi). On November 15, 2025, the second cycle of VP combined with Azacitidine was administered (specific drug dosages and administration as per September 21, 2025). A bone marrow re-examination on December 22, 2025, showed: The bone marrow morphology report (**Figure 1C**) indicated active cellularity with no detectable blasts; the bone marrow immunophenotyping (flow cytometry) report (**Figure 6**) revealed CD34+CD117+ myeloid blasts accounting for approximately 0.22% of nucleated cells, with an abnormal immunophenotype. CBC showed: WBC: 21.49 × 10^9^/L, Absolute neutrophil count: 16.66 × 10^9^/L, Absolute monocyte count: 3.03 × 10^9^/L, Hb: 118 g/L, PLT: 61.00 × 10^9^/L. Given that the peripheral platelet count had not normalized and flow cytometry detected residual minimal blasts, to help the patient achieve a deep and durable CR, we initiated chemotherapy with the VP regimen combined with the VA regimen on December 23, 2025 and January 28, 2026 respectively, specifically: Vincristine 1.4 mg/m² weekly + Prednisone 1 mg/kg + Azacitidine 100 mg on days 1-7 + Venetoclax 100 mg on day 1, 200 mg on day 2, and 400 mg from day 3 to day 14. As the patient experienced systemic weakness symptoms when the Venetoclax dose was increased to 400 mg, with significant adverse effects and intolerability, Venetoclax was administered at 100 mg on days 1-8.

**Figure 7:**
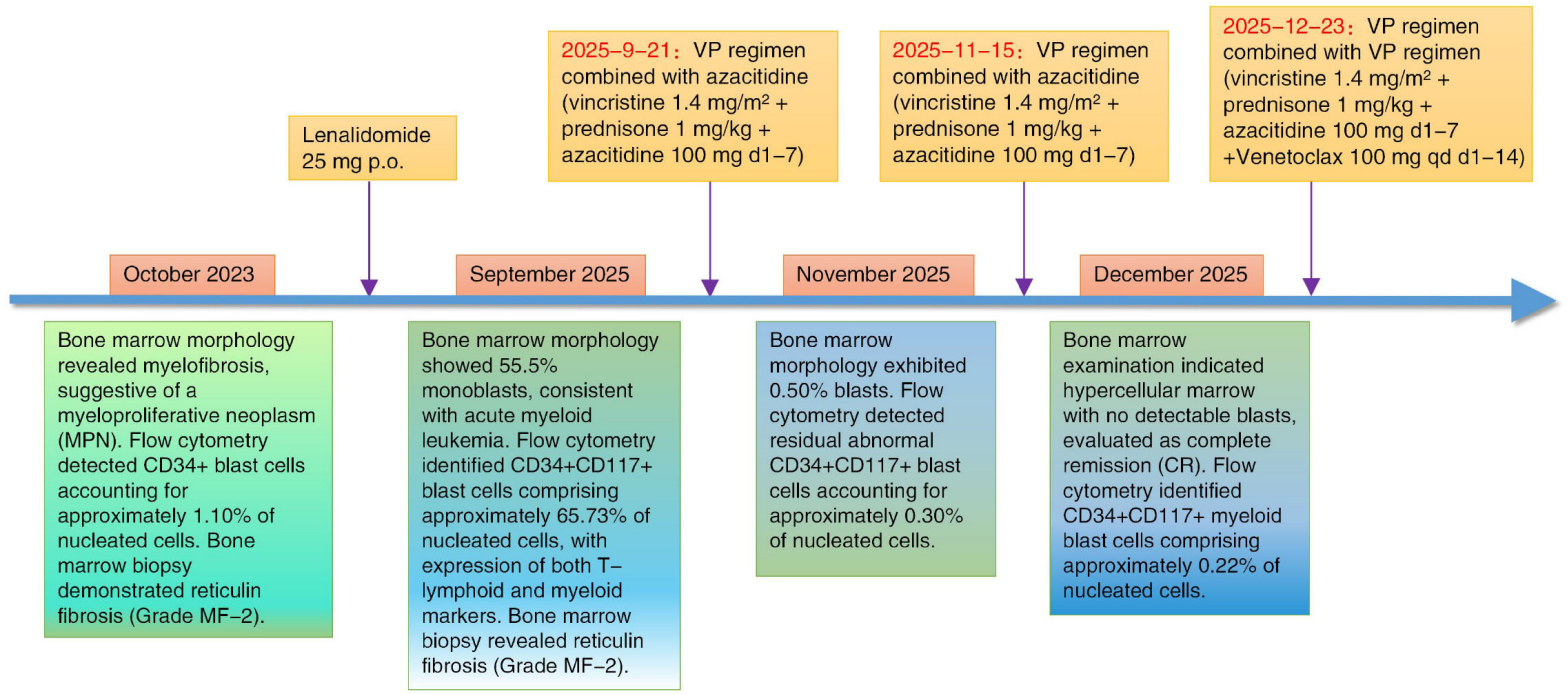
The treatment process diagram of the patient with MPAL transformed from PMF.

As of the latest follow-up conducted on February 28, 2026, this patient is currently in good general condition, able to perform normal physical and social activities, with acceptable appetite and sleep. She is adhering to regular follow-up visits after discharge. Relevant laboratory results remain within a safe range, and no peripheral blasts have been detected. Her physical and mental status is satisfactory, she reports no specific discomfort, and her disease appears well-controlled. The treatment and diagnosis process of the patient in this study is detailed in **Figure 7**. The clinical data and follow-up records of this patient have also been meticulously recorded in **Table 1**.

**Table 1:** Clinical data and follow-up records of the patient’s subsequent hospital visits following transformation from PMF to MPAL.

Time point	Laboratory findings	Bone marrow examination	Specific treatment regimen	Adverse events (CTCAE v5.0)	Transfusion requirements	ECOG performance status
2025-9	Peripheral blasts: 64%, WBC: 47.14×10^9^/L, ANC: 8.78×10^9^/L, Hb: 131 g/L, PLT: 41×10^9^/L	BM morphology: 55.5% monoblasts. Flow cytometry: CD34+CD117+ blasts accounting for 65.73% of nucleated cells, co-expressing T-lymphoid and myeloid markers.	VP + Azacitidine chemotherapy	Febrile neutropenia, Grade 4 Thrombocytopenia, Grade 4 Anemia, Grade 3	Five therapeutic doses of apheresis platelets	3 scores
2025-11	No peripheral blasts, WBC: 10.28×10^9^/L, ANC: 6.85×10^9^/L, Hb: 68 g/L, PLT: 45×10^9^/L	BM morphology: markedly active hyperplasia, 0.50% blasts. Flow cytometry: residual abnormal CD34+CD117+ blasts accounting for 0.30% of nucleated cells.	VP + Azacitidine chemotherapy	Numbness in hands and feet, Grade 2 Thrombocytopenia, Grade 2	None	1 score
2025-12	No peripheral blasts, WBC: 21.49×10^9^/L, ANC: 16.66×10^9^/L, Hb: 118 g/L, PLT: 61.00×10^9^/L	BM morphology: active hyperplasia, no blasts seen. Flow cytometry: CD34+CD117+ myeloid blasts accounting for 0.22% of nucleated cells.	VP + VA chemotherapy	Numbness in hands and feet, Grade 2 Thrombocytopenia, Grade 2	None	1 score
2026-01	No peripheral blasts, WBC: 16.31×10^9^/L, ANC: 11.45×10^9^/L, Hb: 133 g/L, PLT: 65.00×10^9^/L	BM morphology: active hyperplasia, no blasts seen. Flow cytometry: CD34+ blasts accounting for 0.02% of nucleated cells.	VP + VA chemotherapy	Numbness in hands and feet, Grade 2 Thrombocytopenia, Grade 2	None	1 score

PMF, primary myelofibrosis; MPAL, mixed phenotype acute leukemia; WBC, white blood cell count; ANC, absolute neutrophil count; Hb, hemoglobin; PLT, platelet count; VP, regimen containing vincristine and prednisone; VA, regimen containing venetoclax and azacitidine; CTCAE, Common Terminology Criteria for Adverse Events; ECOG, Eastern Cooperative Oncology Group.

## 3 Discussion

This article reports a rare case of transformation from primary myelofibrosis (PMF) to mixed phenotype acute leukemia (MPAL) in a 75-year-old female patient. This case not only comprehensively illustrates the natural history and clinical challenges of the evolution from a chronic myeloproliferative neoplasm (MPN) to a high-risk acute leukemia, but also provides valuable clinical insights into the management of end-stage events in myelofibrosis (MF) and the diagnosis and treatment of MPAL, due to its unique transformed phenotype, complex molecular background, and the therapeutic dilemmas associated with advanced age.

### 3.1 From MF to MPAL: a rare clonal evolution with an poor prognosis

Transformation to acute leukemia is the most severe complication of MF, occurring in approximately 10%-20% of cases, and typically manifests as acute myeloid leukemia (AML) (10–12). However, transformation to MPAL, which co-expresses myeloid and lymphoid antigens, is exceedingly rare. In this case, at the time of disease progression in September 2025, bone marrow flow cytometry definitively revealed blasts co-expressing CD34, CD117, CD3+, CD7, and other markers, meeting the diagnostic criteria for MPAL (T/Myeloid mixed phenotype) (13). The rarity of this transformation event underscores the atypical nature of its clonal evolution. At initial diagnosis, the patient harbored the typical MPN driver mutation J*AK2 ^V617F^*, accompanied by an *ASXL1* mutation (a molecular marker of poor prognosis in MF). Upon transformation, in addition to the persistence of the original mutations, additional mutations including *NRAS*, *EZH2*, and *TET2* emerged. This evolution of the mutational landscape – from “MPN-characteristic mutations” (*JAK2*, *ASXL1*) to the acquisition of “leukemia transformation-associated mutations” (e.g., activating NRAS mutation, associated with sustained proliferative signaling and chemotherapy resistance; *EZH2* involved in epigenetic dysregulation; *TET2* implicated in differentiation block) – delineates a classic molecular pathway from chronic clonal hematopoiesis to highly aggressive acute leukemia. This case suggests that in MF patients, even if MPAL is not the initial presentation, the malignant clone may possess the genetic instability to develop multi-lineage differentiation potential, ultimately leading to an extremely complex phenotype.

### 3.2 Diagnostic and therapeutic dilemmas in the context of advanced age and comorbidities

This case epitomizes the unique challenges faced by elderly patients with hematologic malignancies. Firstly, diagnostic adherence and continuity of care are paramount. Following the initial diagnosis, the patient declined standard targeted therapy with ruxolitinib due to financial constraints and failed to adhere to regular follow-up, potentially leading to missed opportunities for monitoring early signs of disease progression. She did not present again until marked leukocytosis (47.14 × 10^9^/L) and a surge in peripheral blood blasts (64%) had occurred, by which time the disease had already advanced to an acute leukemia phase, forfeiting a potential window for intervention during the accelerated phase. Secondly, treatment options are extremely limited and often ineffective. At the time of MPAL diagnosis, the patient was 75 years old with concurrent, persistent MF (Grade 2 myelofibrosis), resulting in poor bone marrow reserve. This precluded the possibility of receiving standard intensive induction chemotherapy. Our clinical team’s choice of the “VP (Vincristine + Prednisone) combined with a hypomethylating agent (Azacitidine)” regimen represents a combination of low-intensity chemotherapy and epigenetic modulation, often used in elderly or unfit AML patients intolerant to intensive therapy. Although initial treatment achieved hematologic remission (CRi), the remission was exceedingly short-lived, with rapid recurrence observed during the interval between chemotherapy cycles. A subsequent attempt to incorporate the BCL-2 inhibitor Venetoclax was also limited by the patient’s inability to tolerate the treatment-related toxicity, preventing the administration of a full dose and course.

This treatment course clearly reveals that for MPAL transformed from MF, especially in elderly patients, existing low-intensity regimens have limited and unsustainable efficacy, making disease control exceptionally difficult and leading to an poor prognosis. However, it is encouraging to note that this elderly female patient achieved CR of the bone marrow after undergoing two courses of the VP combined with azacitidine regimen and one course of the VP combined with VA regimen. Moreover, her mental, psychological and physiological conditions have recovered well. This once again demonstrates the high heterogeneity and individual variability of such hematological malignancies. It is noteworthy that, although the elderly female patient in this case study achieved an MRD-negative CRi status as assessed by bone marrow morphology, flow cytometry, and other detection methods, the follow-up period was relatively short. Furthermore, due to financial constraints, the patient was unable to undergo follow-up molecular biological testing, meaning the depth of remission at the molecular level could not be confirmed. We acknowledge this limitation and emphasize the need for long-term follow-up to evaluate the durability and stability of the treatment efficacy.

### 3.3 Unique insights and clinical significance of this rare case

#### 3.3.1 Implications for monitoring MF patients

This case strongly underscores the necessity of long-term, regular follow-up for MF patients, particularly those harboring adverse prognostic mutations like *ASXL1*. Monitoring should extend beyond routine blood counts and spleen size; prompt repeat bone marrow examination with MICM classification is crucial whenever transformation is clinically suspected, as the transformation may not be typical AML but the more challenging MPAL.

#### 3.3.2 Complexity of MPAL diagnosis

The transformed leukemic cells in this case co-expressed myeloid and T-lineage markers, necessitating precise immunophenotyping via multiparameter flow cytometry for accurate diagnosis. Relying solely on morphology could easily lead to a misdiagnosis of AML, potentially overlooking critical lymphoid antigen expression that might influence therapeutic strategy (although no standardized regimen exists for MPAL, phenotypic information holds potential value for exploring targeted therapies).

#### 3.3.3 Re-evaluation and exploration of therapeutic strategies

The treatment response pattern in this case — initial efficacy followed by rapid drug resistance and relapse — suggests that MPAL leukemic stem cells arising from an MF background may possess enhanced drug resistance and a survival advantage. For such patients, low-intensity chemotherapy or hypomethylating agents alone may be insufficient to eradicate the malignant clone. Future strategies may need to explore personalized approaches based on molecular characteristics, such as considering combining a *JAK2* inhibitor (ruxolitinib) with a hypomethylating agent or other novel targeted agents (e.g., directed at specific signaling pathways), if tolerated, to target both the MF and leukemic clones simultaneously. However, advanced age and performance status remain the most significant limiting factors.

#### 3.3.4 Supportive care and prognostic expectations

For elderly MPAL patients transformed from MF, while active treatment attempts are made, early integration of palliative care principles is necessary to balance anti-tumor therapy with quality of life, define clear treatment goals (whether aiming for remission versus disease control and symptom improvement), and communicate these effectively with the patient and family.

### 3.4 Recent advances in the treatment of MPAL

MPAL is classified under the broader category of acute leukemias of ambiguous lineage in both the World Health Organization’s Classification of Haematolymphoid Tumours (5th edition) (WHO-HAEM5) and the 2022 International Consensus Classification (ICC) (14, 15). To date, standardized treatment guidelines for MPAL remain lacking, and there is no unified consensus on therapeutic strategies. Huang et al. (16) reported a successful case of an new-diagnosed elderly patient with early T-cell precursor (ETP)/myeloid MPAL treated with venetoclax/azacitidine. This regimen successfully achieved CR without adverse side effects. Concurrently, relevant studies (17, 18) have found that patients with newly diagnosed MPAL and those with relapsed/refractory AML achieve promising responses following VEN-HMA regimens (venetoclax + hypomethylating agents). Furthermore, the “chemo-free” concept emphasized in recent years is gradually gaining traction in MPAL. Liu et al. (19) demonstrated that for B/T MPAL patients with a complex karyotype, a combination regimen of venetoclax, azacitidine, and blinatumomab can achieve both hematologic and molecular complete remission without significant organ or hematologic toxicity. Similarly, Wang et al. (20) confirmed that a chemotherapy-free regimen (venetoclax, a hypomethylating agent, and blinatumomab) can successfully treat patients with B-lymphoid/myeloid MPAL, achieving CR and representing a safe and effective treatment approach. Notably, recent research (21) indicates that low-intensity induction regimens containing venetoclax yield high remission rates, good tolerability, and low early mortality in newly diagnosed adult MPAL, facilitating successful bridging to allogeneic hematopoietic stem cell transplantation. This suggests a potential paradigm shift in MPAL treatment strategies toward “low-intensity, targeted” approaches and also highlights the potential value of targeted agents (e.g., venetoclax, blinatumomab, TKIs) in MPAL. Of significant interest, a systematic review by Karasek et al. (22) outlines that MPAL treatment encompasses strategies and modalities such as Tyrosine Kinase Inhibitors, FLT3 Inhibitors, T-Cell Engaging Therapies, Phenotype-Directed Therapy, DOT1L, Menin, or Bromodomain Inhibitors, and Signaling Pathway Inhibitors, alongside hematopoietic stem cell transplantation. However, clinical application urgently requires personalized treatment strategies based on molecular characteristics, and the conduct of prospective multicenter trials is also critically important for MPAL therapy.

## 4 Conclusion

In summary, this case of a 75-year-old female patient with PMF transforming to MPAL serves as a vivid model demonstrating the complex evolution of a malignant bone marrow clone and the diagnostic and therapeutic dilemmas encountered in elderly patients. It alerts clinicians to maintain a high index of suspicion for leukemic transformation in MF patients and to recognize that the transformed phenotype can be a rare mixed phenotype. Therapeutically, it highlights the urgent need to develop more effective and better-tolerated therapies for this specific patient population. Future research should focus on elucidating the precise molecular mechanisms driving MF-to-MPAL transformation and, based on these insights, design combination targeted strategies to improve the clinical outcomes for this group of patients with an poor prognosis.

## Data Availability

The original contributions presented in the study are included in the article/supplementary material. Further inquiries can be directed to the corresponding author.
